# Plants Consumption and Liver Health

**DOI:** 10.1155/2015/824185

**Published:** 2015-06-28

**Authors:** Yong-Song Guan, Qing He

**Affiliations:** ^1^Department of Oncology, West China Hospital, Sichuan University, Chengdu 610041, China; ^2^State Key Laboratory of Biotherapy, West China Medical School, Sichuan University, Chengdu 610041, China

## Abstract

The liver is a very important organ with a lot of functions for the host to survive. Dietary components are essential for and can be beneficial or detrimental to the healthy or diseased liver. Plants food is an essential part of the human diet and comprises various compounds which are closely related to liver health. Selected food plants can provide nutritional and medicinal support for liver disease. At the present, the knowledge of the effects of plants on the liver is still incomplete. The most urgent task at the present time is to find the best dietary and medicinal plants for liver health in an endless list of candidates. This review article updates the knowledge about the effects of plants consumption on the health of the liver, putting particular emphasis on the potential beneficial and harmful impact of dietary and medicinal plants on liver function.

## 1. Introduction

As a proverb goes, “a closed mouth catches no flies.” Selecting the best food for the mouth is essential for good health, especially the health of the liver. The liver is the largest digestive gland in the body playing a major role in metabolism of various substances. The liver is also under the great load of conducting various functions for the survival of the host, including detoxification, breakdown of red blood cells and substances, synthesis of proteins and hormones, and storing glycogen, as well as holding a reservoir of blood [1, 2]. Any damage that weakens the functioning of the liver is called liver disease including liver cancer [3, 4]. Currently food of plant origin is consumed more frequently for human health and leafy plants or plant parts are eaten usually as vegetables [5]. Generally speaking, all the plants, plant parts, and their ingredients which we ingest are related to the health of the liver because of the existence of enterohepatic circulation and hepatic detoxification [6].

Dietary and medicinal prevention or treatment of liver disease by plant-based stuff is an essential constituent of complementary and alternative medicine [7]. Human has a long history of consuming edible plants for food and survival and now still consumes a wide variety of wild and semidomesticated food plants, domesticated crops, vegetables, fruits, and plant food supplements, as well as plants for medicinal use [8–11]. In spite of the long history and wide distribution of use, the knowledge of the impact of these plants on the liver remains incomplete [12]. In addition, the known knowledge of botany-hepatology as a discipline can be used to learn new knowledge in the era of molecular medicine, and many of the traditional views and opinions gained through experience need to be confirmed by modern technology on the basis of evidence [13–16]. In a tremendous body of countless plants, finding the best ones with edible or potable elements for liver health presents too much a challenge to the researchers of liver disease. Based on the most recent literatures in the area, this paper reviews the impact of plants consumption on the health of the liver, with special emphasis on the positive and negative influence of dietary and medicinal plants on liver function.

## 2. Wild and Semidomesticated Food Plants Good for Liver

There is a very long history of consumption of domesticated and cultivated food plants including crops, fruits, and vegetables. People should have gained a lot of knowledge about the effects of these foods on human health. For example, the earliest domestication of common millet in East Asia can be dated to the Neolithic era 10,000 years ago [8]. However, both animal and human studies on many of these plants, like berries, carrot, grapes, ginger, green tea, pistachio, pomegranate, tomato, and wheat, have yielded conflicting results; thus, it is now still very hard to recommend what is the best estimate of the amount of these plants a person consumes for liver health [1, 3, 4].

The wild and semidomesticated food plants are now consumed as supplements to the domesticated foods and as main foods to suppress hunger at times of food shortage in underdeveloped world [10]. For example, the Northeast region of Thailand is regarded as the largest and poorest portion of the country. In anthropogenic areas there, wild food plants are a fundamental part of the diet for vulnerable farmer households and acquisition of wild food plants improves the readiness of seasonal crop throughout the year [17]. Forms of wild food plants include aquatic herb, bamboo, climber, rattan, terrestrial herb and tree, and edible parts of these plants cover shoot, flower, fruit, whole plant, leaves, cladode, seed, tuber, rhizome, stalk of flower, and stem [17, 18].

Reduction-oxydation (redox) state represents a crucial background of various liver disorders. There is always the paradox of oxygen use in metabolism for the existence of life. On one hand, oxygen is fundamental for the organism to survive. On the other hand, oxygen as a strong reactive molecule devastates the organism by generating reactive oxygen species. The organism in turn develops an antioxidant network to prevent this damage. Oxidation reaction can generate free radicals which can damage cell membranes and cause diseases. The imbalance between production of reactive oxygen species and the system's defense represents oxidative stress which is one of the essential pathogenic factors in numerous liver diseases including inflammatory, metabolic and proliferative ones and antioxidants are chemicals rich in many food plants and can be used as prevention and treatment of such diseases [19]. Almost all chronic liver diseases are under the background of elevated oxidative stress. The organism maintains various systems of antioxidants which could be simply divided into enzymatic and nonenzymatic categories (Table 1).

Great sources of essential antioxidants are foods rich in vitamin C, vitamin E, and trace element selenium [1]. Some nonessential substances from plant food origin also have antioxidant activity, such as ascorbic acid, *β*-carotene, coenzyme Q10, curcumin, dong quai (*Angelica sinensis*), ebselen, ellagic acid, epigallocatechin gallate, lipoic acid, lycopene, mitoquinone, N-acetyl cysteine, quercetin, and resveratrol [20–23]. Minor dietary nonnutrients in plant foods also have notable activity in cancer prevention by their effects of suppression or block or both on carcinogenesis. Inhibitors with suppressing effect prevent development of tumorigenesis in cells that would become cancerous under other circumstances while those with blocking effect prevent cancer-causing agents from contacting or responding with all-important target sites [3, 24].

Redox state activates the innate immune system and elevates the discharge of proinflammatory cytokines and other mediators for the establishment of alcoholic liver disease and nonalcoholic fatty liver disease. And oxidative stress also encourages the progression from steatosis to steatohepatitis [25]. Redox state impacts on brain function in hepatic encephalopathy by overproduction of reactive oxygen and nitrogen oxide species [26]. Fibroproliferative liver diseases can result from disturbance of redox homeostasis by activation of myofibroblast-like, hepatic stellate cells, and other profibrogenic cells [27]. Animal and human studies have shown that both hepatitis B virus (HBV) and hepatitis C virus (HCV) stimulate oxidative stress in liver cells with elevated oxidative DNA damage. In addition, both viruses disturb the response of liver cells to oxidative DNA damage with consequent genome instability and cancer formation [28]. Several RNA viruses including HCV are reported to induce oxidative stress with changes in host defense modulated by antioxidants [29].

Phytoestrogens are dietary plant estrogens that are not produced in the endocrine system but obtained by consuming phytoestrogenic plants. These nonsteroidal plant compounds are naturally occurring and similar in structure to estradiol with estrogenic and/or nonestrogenic effects exerted by binding to estrogen receptors. A group of phytoestrogens in the Coumestan family has been proved to be anti-HCV agents by inhibiting viral RNA replication [30]. One of the phytoestrogens that is called genistein falls into the category of isoflavones and has been demonstrated at the molecular level to have similar therapeutic effect to interferon-*α* on HBV infection [31].

Plant foods richest in phytoestrogens are oilseeds and nuts. The most frequently encountered sources of phytoestrogens are the plants of the legume family [32]. Such plant foods from wild and semidomesticated origin include alfalfa, anise, chaste-tree berry,* Dunbaria villosa*, fennel, fenugreek, ginkgo, ginseng, hops, kava, kudzu, lentil, licorice root, lupine, mint, psoralea, red clover, saw palmetto, and wild yam [33, 34]. Nevertheless, evidence is lacking for therapeutic use of these plants, and clinical trials are needed with concerns for long-term safety and efficacy.

Other hepatoprotective plant foods from wild and semidomesticated origin consist of amaranth,* Aralia elata Seem*, asparagus, balloonflower root (*Platycodon grandiflorus*), buckwheat, capillary wormwood, celery, chestnut, Chinese chive, Chinese small iris* (Iris lactea)*, Chinese toon, heartleaf (*Cordate houttuynia*), cress, dandelion, daylily, devil's tongue (*Lilium brownii*), hawthorn, hazelnut, kelp, kiwi fruit, longan, longstamen onion bulb, lotus root, mango, Manyflower Gueldenstaedtid herb (*Herba Gueldenstaedtiae*), olive, papaya, philippine violet herb (*Herba violae*), purslane, red date, rivier (*Rhizoma amorphophalli*), shepherd purse, sow thistle, spring bamboo shoots, summer squash, tangerine, tzu tsai (*Porphyra haitanensis*), wild bracken, and yam [35–39]. The list is still growing and in-depth studies on different phytonutrients are warranted for rationale consumption of these plant foods to improve liver health. Well-designed randomized clinical trials are needed.

## 3. Wild and Semidomesticated Food Plants Harmful to Liver

Phytoestrogens are now used for estrogen replacement as complementary and alternative therapy of several conditions. For example, black cohosh is widely advertised as bust-enhancing product and prescribed for menopausal symptoms and pain relief. However, this product has been associated with liver toxicity [40]. Many of the dietary plants associated with phytoestrogens are substrates for a genus of fungi called Fusarium to produce zearalenone which is a potent estrogen and has strong genotoxicity and liver toxicity [34, 40]. Excessive phytoestrogens have adverse effects not only on the reproductive system but also on the liver [41]. Use of food containing phytoestrogens is generally safe. However, estrogen-like effects are observed and increased with prolonged use [42]. A safety study on a phytoestrogen called genistein in Wistar rats demonstrated that very slight proliferation of bile duct, increased gamma glutamyl transferase, and hypertrophy of liver cells were observed at repeated doses of 500 mg/kg/day [43]. Another study of genistein effects on Wistar rats found that the phytoestrogen has strong impact on hepatic gene expression [44]. Phytoestrogens also have been shown to induce gene activation in human liver hepatocellular carcinoma cell line HepG2 cells [45]. So, researches are needed to acquire knowledge of avoiding particular components of plant-based foods for liver health.

Pesticide residue in wild plant foods is highly hepatotoxic and leads to change in metabolism and oxidative balance in the liver [46]. Fumigated or grilled foods are also harmful. A study showed that fumigation residues bound on seeds were highly bioavailable to experimentally fed animals with resultant hepatic injury [47]. Animals that were fed on a diet consisting of grilled foods showed with elevated serum levels of cholesterol, aspartate transaminase, creatinine, and urea and many kinds of chromosomal aberrations in examined cells [48]. Foods can go bad easily in summer. Deteriorated and rotten foods are full of molds and fungi and are dangerous to eat. One study examined the effects of decayed foods on the liver in rats. Male Wistar rats were fed with a diet containing gluten thermally processed with oil spontaneously for 11 weeks and damage in the liver occurred subsequently [49]. Rotten ginger is strongly poisonous. Rotting ginger produces a highly toxic substance safrole known as natural hepatoxin which leads to liver cell degeneration and necrosis and may induce liver cancer as tumorigenic effects in the liver were shown after long-term exposure of animals to other plants [50, 51]. Rotten potatoes as well as other food plants also have potent toxic effects on the liver through the intake of mycotoxins because rotting plants are frequently infected with* Fusarium spp.* [33, 40, 52].

Cassava is a woody shrub and is widely consumed for its starchy tuberous root as food in Latin American, Caribbean, African, and Asian countries. It is the third major source of dietary carbohydrates in the tropical zone, following rice and maize. However, people consume cassava excessively or incorrectly are at risk of poisoning. Goats fed with cassava leaves for 30 consecutive days showed toxic effects of cyanogenic glycosides with vacuolation of periportal hepatocytes [53]. Liver cancer has also been associated with this food plant [54, 55].

Currently literature is limited about liver damage induced by food plants but evidence will continue to accumulate for the effects of dietary components on the liver. The potential hazards of nightshades to liver health are described in the next section.

## 4. Medicinal Use of Food Plants for Liver Health

For thousands of years people have the belief of food as medicine and medicine as food [56]. A commonest English axiom reads, “an apple a day keeps the doctor away.” Apples were one of the earliest foods that medical specialists accepted as beneficial and healthy. Apple polyphenol extract has been shown to have hepatoprotective effects on liver oxidative stress which was induced by aluminum chloride in the rat [57]. Tamoxifen is a nonsteroidal antiestrogen and has been used to induce oxidative stress in rats showing increase in aminotransferases. This effect was reduced significantly by a food product consisting of dried apple and mandarin juice [58].  Table 2 briefly lists medicinal use of common plant foods for liver health.

Green leaves are the best for liver health. There is the saying in traditional Chinese medicine: “the dark-green colored falls into liver meridian.” A flavone glucoside named as saponarin has been extracted from young green barley leaves. This flavonoid gives the typical green color to the leaves and demonstrates powerful antioxidant potencies with therapeutic effects on various cancers and inflammations [59].* In vivo* studies proved that green tea leaves have strong inhibitory activity for liver cancer. Camella sinensis is a common Chinese green tea. Alcoholic extract of the leaves of this plant was prepared and given by gavage to Wistar rats bearing Walker-256 liver cancer. Strong antitumor activity was achieved in rats that received the treatment with the green tea extract [60].

Several categories of food plants have chemopreventive effects on carcinogen-induced neoplasia. They are cruciferous vegetables, citrus fruits, caraway (*Carum carvi*) seed oils, and* Allium* species [4, 61]. Cruciferous vegetables are green leafy veggies including bok choy, broccoli, cabbage, cauliflower, and cress. A large integrated series of case-control studies consisting of 1468 cancers presented supporting evidence of favorable effect of these food plants on several common cancers [62]. Citrus is the general name for many flowering plants cultivated since ancient time. The well-known citrus fruits are the grapefruit, lemons, limes, mandarins, and oranges. Citrus fruit oil was reported to improve hepatotoxicity in chickens fed with a diet containing aflatoxin, a potent hepatocarcinogen, showing reduced lesions of hydropic degeneration and bile duct hyperplasia in the liver [63]. Caraway is also called meridian fennel and has long been used as a valuable aromatic herb and a spice in food to enhance flavors. This plant shows a large range of antimicrobial activities especially distinct inhibitory effects on growth of fungi and aflatoxin production. Caraway seed oils are commonly employed as household medicine for many ailments including hepatobiliary complications [64].* Allium* is the term for garlic in Latin language and represented unofficially as the onion genus. Food plants in the* Allium* genus include different chives, garlics, leeks, onions and scallions. They present various flavors and mouthfeels and are consumed either cooked or raw all over the world in different delicacies. A number of studies both* in vitro* and* in vivo* have been published reporting that allium-genus plants have potent hepatoprotective activity and distinct effects on various liver conditions such as hypercholesterolemia-induced oxidative stress, cadmium liver accumulation, liver fibrosis, liver fluke, and alcoholic fatty liver [65–69].

Patients with liver disease are advised to avoid nightshade plants which are the common name for the Solanaceae family that consists of more than 2800 plants. Well-known nightshades include eggplant, ground cherries (any of the genus* Physalis*), mandrake (*Mandragora officinarum*), peppers, pimentos (*Capsicum annuum*), potatoes, tobacco, tomatillos (*Physalis ixocarpa*), and tomatoes. Animal study of several nightshades resulted in the conclusion that the plants are hepatotoxic showing amyloidosis and moderate necrosis in liver [70]. Another nightshade plant (*Solanum cernuum Vellozo*) was also involved in hepatic toxicity when it was used in high dose and significant increase in the activities of alanine aminotransferase and aspartate aminotransferase was observed [71]. Jimson weed (*Datura stramonium*), also known as thorn apple, is a nightshade plant having spiny capsule fruits. Jimsonweed is ingested by some people to enjoy hallucinations that this plant can cause. However, jimsonweed is strongly poisonous and sometimes fatal. Jimsonweed intoxication can lead to fulminant hepatitis and acute liver failure requiring subsequent liver transplantation for salvage [72, 73].

Manufacturers of dietary supplements usually cannot provide clinical data supporting their claims of safety or efficacy [16]. Physicians and the general public must take care of drug-food interactions and potential adverse effects when plant-based foods are used for medicinal purpose [74]. A physician must know as more as possible pharmacokinetic interactions of phytochemicals with drugs although currently our knowledge about nutrient-drug interactions is still limited and efforts to elucidate them should be reinforced [75]. Laxative plants can be used to clear the ingested toxins away from the digestive system. Such plants include aloe vera, dandelion, rhubarb rhizome and senna leaf [76]. The mung bean or moong bean is also known as green gram and is regarded as a detoxification agent for thousands of years in both in Traditional and folk Chinese medicine. Mung bean accelerates metabolism and transformation of toxins in food and drug by special enzymes which involve in the biosynthesis of phenolic compounds. Mung bean sprout produces several kinds of hepatoprotective compounds such as flavonoid and chlorogenic acid [77]. An aldehyde reductase has been extracted from mung bean that detoxifies fungal toxins [78]. Radish is in the Cruciferae family and also known as “Laifu” or “Luobo” in Chinese that has a history of being used for medicinal purpose for more than a thousand years. Spanish black radish comprises unique glucoraphasatin which has been proved to be a potent inducer of detoxification enzymes in liver cancer cell [79]. The degradation products of glucoraphasatin, such as sulforaphene, raphasatin and glucoraphenin, are also liver detoxification enzymes although not as potent as glucoraphasatin [80].

## 5. Liver Disease Herbs for Specific Therapy

*Phyllanthus urinaria* is an herbal medicine with potential antioxidative properties and has been proved to improve steatohepatitis both in cell cultures and in mice, perhaps via decreasing oxidative stress, relieving inflammation, and reducing lipid accumulation [14]. A trial of 1145 participants examined the effects of silymarin, an herbal product extracted from milk thistle (*Silybum marianum*), on patients with advanced chronic hepatitis C. The results showed that use of silymarin had no effects on hepatitis C virus RNA levels or serum alanine aminotransferase, but that better quality-of-life indices and fewer hepatic symptoms were observed in the silymarin users [15]. Curcuminoids have been proved to safeguard DNA against reactive oxygen species and protect liver cells in the time of liver damage and cirrhosis [81].

Simaroubaceae (*Picrasma quassioides*) is a family of tropical trees and shrubs and has been shown to be protective for carbon tetrachloride-induced liver injury and effective in treating liver cancer in moderate and late stage by comprehensive Chinese medicine with extended pain-relieving sustained time, improved quality-of-life, prolonged survival, and less adverse effects [82].

There are a great variety of live disease herbs all over the world and about 80% of the world population use herbal medicine. Elucidation of medicinal properties and hepatoprotective compounds of these herbs is of principal significance [83]. Herb induced adverse effects on liver functions are called herbal hepatotoxicity and there have been public concerns of the use of herbs. Although a large number of herbs and plant products have been involved in the causation of liver injury, the majority of the issues in causality is not yet validated and lack of enough evidence [13]. The establishment of the definite diagnosis for toxic liver disease needs hard evidence or at least sufficient supporting evidence. These reports often fall short of necessary diagnostic details and the methods for evaluating causality are nonspecific. Many external factors, such as difference in batches, adulterants, impurities, and misidentification of plant species, lead to the negative results of assessment [13]. The judgment system of herbal hepatotoxicity has to be improved for future research.

## 6. Amount and Methods of Consumption and Combination Use of Plants

It is of paramount importance to eat a well-balanced diet for liver health. A recent study conducted in Japan investigated the effects of well-balanced lunches on liver function. The lunch was low in animal protein and high in vegetables. This diet was provided to 10 subjects once a day for 1 month. At last, serum alanine aminotransferase status of the subjects reduced by 20.3% [84]. Although we are talking about plants consumption here, the role of animal protein and fat in improving liver health cannot be neglected and the amount of plant components in the food should be well regulated as a diet rich in sucrose was shown to cause inflammation and liver damage in mice [85]. The oxidative efficiency declines along with the age growth of the individual and consequently some chemical substances that need oxidation might accumulate to cause toxicity [86]. Simultaneous use of drugs with herbs may imitate, intensify, or counter the effect of drugs resulting in herb-drug interactions, and prediction and identification of such interactions present challenges for health professionals involved in the management of liver disease [50, 87].

*Coleus forskohlii* extract is a natural herbal product commonly used to offset obesity and induces hepatic drug metabolizing enzymes [12]. This induction is enhanced in mice by the amount of dietary starch, implying that the combination of* coleus forskohlii* extract and food rich in starch further increases enzyme activity of the liver. Hepatotoxicity of this plant is dose-related and hepatic cytochrome P450 enzyme is induced significantly [88].

The processing method of plant food is usually the key point for liver health [89]. For example, brown rice is well known as a healthy food. However, the therapeutic effect will be highly decreased when brown rice is simply steamed or cooked over for a patient with liver disease to take. The best way of processing it is preparing rice gruel, a very thin porridge that has been known as “congee” and applied in China for thousands of years to the treatment of digestive diseases. Brown rice congee is easy to digest and helpful for the liver to recover naturally [90]. But this procedure demands time, patience, and skill. Laba porridge, also known as babao gruel, is highly nutritious and famous meal eaten on the day that by folk legend Prince Siddhartha attained the top of enlightenment and became Buddha after eating this congee [91]. Babao means eight treasures and is made of eight elements. Today there are dozens of recipes for this dish using various nutritious and therapeutic ingredients such as glutinous rice, red bean, mung bean, black soybean, peanut kernel, sorghum, foxtail millet, brown rice, red date, Job's tears, lotus seed, lily bulb, and raisin. Each of the components represents a different medicinal use and many of them have hepatoprotective activities [92, 93]. There are several different ways in which plants are prepared and used for therapeutic purpose like eating fresh raw plant, boiling, steaming, sauteing, pickling, oven curing, country curing, solar drying, shade drying and mechanical drying. There are remarkable differences between the methods of preparing plants in effects on liver health. For example, broccoli is best cooked by lightly steaming or stir-frying to preserve the most of its natural nutrients, while boiling or brewing will let the most important nutrients to come into the cooking fluid [21, 75, 89].

Although nightshades show antitumor activity, the mild hepatotoxicity is of concern as stated above. The effects of concomitant consumption of several nightshades on the liver need investigating. Poisoning by black nightshades is of particular concern because a recent investigation from New Zealand listed this plant as the commonest one in the 15 common poisonous plants [94].

The liver can be detoxified and cleansed by drinking more water. Good hydration is important for most basic physiological functions of the liver. Sufficient hydration is required to promote blood circulation and to dissolve nutrients. An adequate intake of water encourages metabolism and facilitates biliary secretion of bile, the process of digestion, absorption, and excretion of wastes to reduce the impairment of the liver by poisoning metabolites and toxins [95]. However, drinking too much water has negative impact on the liver and can be lethal. Acute hypoosmolarity leads to protein conservation related to impaired insulin sensitivity to glucose metabolism, increased lipid oxidation, lipolysis, and ketogenesis [96].

A well life-style is the best medicine. A balanced diet is necessary and binge overeating or frequent starvation should be avoided. Imbalanced food habits result in abnormal secretion of digestive juices and hepatic dysfunction [97]. Good sleep is also very important for liver health. Patients with cirrhosis often have sleep-wake abnormalities and they are observed sleeping significantly less well than healthy subjects [98]. A recent study from Seoul investigated the association of sleep quality and duration with nonalcoholic fatty liver disease in middle-aged males and females. Confounding factors such as alcohol drink and smoking were excluded from the cohort. Poor sleep quality and short sleep duration significantly increased the incidence of this liver disease [99]. Obstructive sleep apnea is now associated with liver injury that is caused by intermittent hypoxia. So sleep on the left or right side may be better for liver health than on the back [100]. Good temper is another great medicine for liver disease. The Traditional Chinese Medicine believes that anger leads to troubles of the liver. Acute stress showed significant impacts on gene expression and function of the liver in the rat, proving “raged impairing liver” [101]. Findings of rage experiences were obtained in HCV patients with evidence showing greater rage was in relation to poorer quality-of-life as negative feedback of the disease [102]. Patients with liver disease are suggested to take limited spicy food. Although curcumin is regarded as a great hepatoprotective product and has now been used in many countries as a supplement, and dietary spice turmeric has been consumed for medicative use for thousands of years, the natural turmeric has been found to contain up to 200 compounds of which the most are toxigenic. In addition, curcumin and its by-products may generate dose dependent hepatotoxicity [103].

## 7. Conclusions

First, do no harm. Various plants are consumed for dietary and medicinal use as wild, semidomesticated, and cultivated crops, vegetables, fruits, and herbs. It is necessary to increase availability of plants safety data to the general public and medical professionals. Safety concerns for plant consumption are rationale because of lack of evidence obtained from well-designed randomized clinical trials for long-term and large amount use. Herbal hepatotoxicity has been reported many a time involving a large number of herbs and plant products, but the most of the causal relationships are not confirmed and lack of convincing evidence.

A well-balanced diet is critically important for liver health. A healthy life-style includes additionally rejoicing with a merry mind, keeping smoke-free and alcohol-free, having good sleep, and drinking adequate water. The intake amount of a certain plant, method of consumption, and combination of plants could be either hepatoprotective or hepatotoxic. Older age should be considered as a risk factor for accumulated toxicity caused by plant chemicals. Concomitant use of drugs and herbs sometimes leads to herb-drug interactions.

Hepatoprotective plants contain substances with antioxidant activities. Plant sources of antioxidants are essential nutrients, such as vitamins and trace elements, and some nonessential substances. Plant-based antioxidants have preventive and therapeutic effects on various liver diseases including alcoholic liver disease, nonalcoholic fatty liver disease, fibroproliferative liver disease, viral hepatitis, and liver cancer. Cruciferous vegetables, citrus fruits, caraway seed oils, and* Allium* species are chemopreventive for liver cancer. Dietary plant estrogens are very effective in treating viral hepatitis but some of them are associated with liver toxicity caused by fungi contamination. Fumigated, grilled, pesticide-contaminated, or rotten plant foods should be avoided. Excessive or incorrect consumption of cassava is harmful. Nightshades show mild hepatotoxicity, and poisoning by black nightshades can be lethal and is of particular concern.

Nearly all the foods we consume are associated with the health of the liver. Diets rich in plant ingredients are getting popular now for human health and people are taking supplements from plant origin both in over-the-counter and in prescription form to detoxify and cleanse the liver. Prevention and treatment of liver disease by dietary or herbal method is one of the important components of complementary and alternative medicine. The knowledge of effects of various food plants on liver health is still insufficient. Traditional methods and experiences about use of food plants for treatment of liver disease must be validated by admissible evidence obtained on the basis of modern technology. The immediate challenge is to find the best dietary and medicinal plants for liver health in an infinite list of candidates. All of these topics require further assessment.

## Figures and Tables

**Table 1:** Simple grouping of antioxidants for liver health.

Group	Name	Characteristics	Effective in liver disease
Enzymatic	Catalase	Very common antioxidant	Alcoholic liver disease
	Superoxide dismutase	Both endo- and exogenous	Chronic liver injury
	Peroxidases	Neutralizing hepatotoxins	Hepatotoxicity

Nonenzymatic	Reduced glutathione	Neutralizing hepatotoxins	Hepatotoxicity
	Melatonin	Power very strong, versatile	Nonalcoholic fatty liver disease
	Ebselen	Glutathione peroxidase analog	Alcoholic liver disease
	Vitamin C	Cofactor in enzyme reactions	Viral hepatitis, cirrhosis
	Vitamin A	Enhancing immunity	Cirrhosis, steatohepatitis
	Vitamin E	Richest in flaxseed oil	Hepatitis B, nonalcoholic fatty liver disease
	4-Hydroxynonenal	Cell signal transduction	Alcoholic liver disease
	Malondialdehyde	Potentially mutagenic	Acute liver cell injury

**Table 2:** Medicinal use of common plant foods for liver health.

Category	Common name	Botanical name	Special active elements	Benefits to liver
Vegetables	Beets	*Beta vulgaris *	Betaine	Chloretic
	Broccoli	*Brassica oleracea *	Diindolylmethane, glucoraphanin	Antiviral, anticancer
	Carrots	*Daucus carota *	Beta carotene and other carotenoids	Antioxidative activity
	Collard greens	*Brassica oleracea *	Diindolylmethane, sulforaphane	Anticancer, anti-inflammation
	Kale	*Brassica oleracea *	A group of resins	Lowering cholesterol and fat
	Sweet potato	*Ipomoea batatas *	Beta carotene, fiber	Attenuating liver injury
	Yams	*Dioscorea alata *	Diosgenin	Inhibiting hepatomegaly
	Cabbage	*Brassica oleracea *	Glucosinolates	Countering alcohol, hangover

Fruits	Avocado	*Persea americana *	Adiponectin	Hypolipidemic activity
	Banana	*Musa acuminata *	Pectin	Relieving cirrhosis
	Cherry	*Prunus avium *	Methyl jasmonate	Antioxidant activity
	Fig	*Moraceae ficus *	Fumaric acid, ficin	Antifatty liver action
	Lemon	*Citrus limon *	Naringin, citric acid	Decreasing liver damage
	Papaya	*Carica papaya *	Lycopene, danielone	Antioxidative activity
	Pomegranate	*Punica granatum *	Punicalagins (pomegranate ellagitannins)	Anticancer
	Watermelon	*Citrullus lanatus *	Citrulline, lycopene	Antitoxic, hypoglycemic

Grains	Barley	*Hordeum vulgare *	Caffeic acid, *p*-coumaric acid	Antifatty liver action
	Maize	*Zea mays *	Lutein, linolic acid	Antioxidative activity
	Brown rice	*Oryza sativa *	Anthocyanins, tocopherols	Anti-inflammatory effects
	Oat	*Avena sativa *	Ergothioneine	Antioxidative activity
	Wheat	*Triticum stivum *	Alkylresorcinols, ferulic acid	Increasing lipid metabolism
	Sorghum	*Sorghum bicolor *	*p*-Hydroxybenzaldehyde, methyl ferulate	Antioxidative activity
